# Knowledge and practices related to salt consumption in China: findings from a national representative cross-sectional survey

**DOI:** 10.1038/s41371-023-00861-7

**Published:** 2023-10-19

**Authors:** Wenrong Zhang, Dinesh Neupane, Zhenping Zhao, Bo Jiang, Mei Zhang, Xiao Zhang, Zhengjing Huang, Chun Li, James E. Sharman, Caihong Hu, Pengpeng Ye, Limin Wang

**Affiliations:** 1 National Center for Chronic and Non-Communicable Disease Control and Prevention, Chinese Center for Disease Control and Prevention, Beijing, China; 2 Department of International Health, Johns Hopkins Bloomberg School of Public Health, Johns Hopkins University, Baltimore, MD USA; 3 Menzies Institute for Medical Research, University of Tasmania, Hobart, TAS Australia; 4 Nanjing Center for Disease Control and Prevention, Nanjing, China

**Keywords:** Risk factors, Hypertension, Lifestyle modification

## Abstract

There is limited information on the knowledge and practice of salt-reduction in China. The purpose of this study was to describe the status of the knowledge and practice of salt-reduction among the Chinese population from a nationally representative survey stratified according to hypertension status. The association between hypertensive status and salt-reduction knowledge and practice was calculated using multivariate hierarchical logistic regression adjusting for related confounders. The study included 179,834 participants; 51.7% were women, and the mean age was 44 years. The levels of overall salt-reduction knowledge (7.9%) and practice (37.1%) were low. The percentage of the use of salt-control spoons and low-sodium salt was 10.7% and 12.2%. The aging population (≥60 years) had the lowest levels of salt-reduction knowledge (5.7%) than other age groups (*P* < 0.0001). People living in rural areas (OR = 0.64; 95% CI: 0.51, 0.81) had lower odds of using salt-control spoons. Females (OR = 1.23; 95% CI: 1.10, 1.36) had higher odds of using salt-control spoons. People living in rural areas (OR = 0.48; 95% CI: 0.36, 0.63) had lower odds of using low-sodium salt. Females (OR = 1.26; 95% CI: 1.13, 1.41) and people living in the southern region (OR = 1.43; 95% CI: 1.11, 1.83) had higher odds of using low-sodium salt. Our work highlights the need to promote education related to hypertension, salt-reduction knowledge and methods among the public and the need to strengthen strategies for the popularization of salt-reduction knowledge and practices among males, people living in rural areas, people living in the northern region and the aging population in China.

## Introduction

Hypertension is a leading risk factor for cardiovascular disease and has an increasing prevalence in low- and middle-income countries [1, 2]. In China, the prevalence of hypertension increased from 18.1% in 2004 to 27.5% in 2018 among adults, and the control rate of hypertension was only 11% [3, 4]. High salt intake is a leading risk factor for hypertension [5]. Excessive salt intake could lead to 3 million deaths and 70 million DALYs globally [6]. The World Health Organization (WHO) recommends that each adult consume less than 5 g (<2 g of sodium) of salt per day [7]. The Chinese Dietary Guidelines in 2015 recommend that salt intake should not be more than 6 grams (≤2.35 g sodium)/person/day [8, 9]. However, in 2020, the average daily salt consumption among Chinese adults was 9.3 g (9.6 g in rural areas, 8.9 g in urban areas), far exceeding all recommended maximum intakes [10–12].

Since the 1980s, several initiatives have been conducted to limit salt consumption in parts of China [10]. Recently, “Action on Salt China” has been organized, which is a salt-reduction campaign in six provinces from 2017 to 2021, aiming to reduce salt consumption by 1 g/day at home and 0.5 g/100 g in restaurants by developing six programs. A key focus of this initiative was to increase salt-reduction knowledge and practices [8]. However, there is limited information on the current status of salt-reduction knowledge and practices in Chinese residents, particularly among people with hypertension. The purpose of this study was to determine the level of knowledge and practices of salt-reduction among Chinese adults who participated in a nationally and provincially representative survey.

## Methods

### Study design

The study used data from the cross-sectional survey of the China Chronic Disease and Nutrition Surveillance (CCDNS) commenced in 2015. Details of the study have been published elsewhere [13–16]. In brief, this survey was nationally and provincially representative and was conducted from June 2015 to May 2016 at the 298 national Disease Surveillance Points (DSPs) distributed in the counties/districts in 31 provinces of mainland China. This survey used a multistage, stratified, cluster-randomized sampling design. From the selected 298 DSPs, only eligible residents who were aged 18 years or older and had been living in the DSPs for at least six months within the preceding year before the CCDNS survey were surveyed.

### Sample size

To be nationally and provincially representative, the sample size included 62 stratums (31 provinces * 2 (rural and urban areas)), and the sample size in each stratum was estimated according to the following equation: *N* = ${deff}\frac{{u}^{2}p(1-p)}{{d}^{2}}$ with *u* = 1.96 (confidence level = 95%), *p* = 9.7% (the diabetes prevalence from the 2010 Report on Chronic Disease Risk Factor Surveillance in China), and deff = 3, *d* = 20%*9.7% (relative error = 20%). According to the above parameters and considering a nonresponse rate of 10%, the estimated sample size was 185,000 (62 stratums multiplied by sample size in each stratum divided by the 10% nonresponse rate). During the survey, a total of 189,605 individuals were participated.

All investigators received unified training before starting the survey. The study protocol was approved by the Ethical Committee of the National Center for Chronic and Noncommunicable Disease Control and Prevention (approval number 201519-A). All participants signed informed consent forms.

### Study variables

#### Sociodemographic variables

Participants self-reported basic demographic information, including age, sex, education level, province and occupation. Urban areas were defined as participants living in urban subdistricts and rural areas were defined as participants living in rural townships. The north was defined as participants living in the north of the Qinling-Huaihe and the south was defined as participants living in the south of the Qinling-Huaihe. Education level was classified as 1) primary or less (6 years); 2) secondary school (9 years); 3) high school (12 years); or 4) college and above. Provinces were classified based on whether they had salt-reduction campaigns until 2015. Specifically, the provinces that had salt-reduction campaigns until 2015 were Beijing, Shanghai, Liaoning, Heilongjiang, Jiangsu, Shandong, Henan, Hubei, Hunan, Guangxi and Guizhou provinces [17–19]. Depending on the work characteristics and sources of salt consumption, the occupation was classified into four groups: 1) physical working population; 2) nonphysical working population; 3) students and soldiers; and 4) others.

#### Blood pressure and hypertension category

Systolic and diastolic blood pressure were measured three times by trained physicians using an Omron electronic sphygmomanometer HBP-1300 with an appropriate size cuff on the left arm. Measurements were obtained after five minutes of seated rest in a quiet environment, with a one-minute interval between cuff inflations. The average of the last two measurements was used for analysis. Established hypertension was defined as a self-reported diagnosis by a doctor or self-report of taking antihypertensive drugs within two weeks before the interview. Newly diagnosed hypertension was defined as the average of the last two blood pressure measurements recorded as systolic blood pressure ≥140 mmHg and/or diastolic blood pressure ≥90 mmHg and not being diagnosed with hypertension or taking antihypertensive drugs within two weeks of the interview [20].

#### Salt-reduction knowledge and practices

Salt-reduction knowledge was defined as knowing that one’s daily salt intake should be no more than 6 grams/person/day, as recommended by the third version of the Chinese Dietary Guidelines (2007) that is the latest version published before the survey. Participants were asked the following question: “How much is the maximum daily salt intake per day for an adult?”. If the respondent gave the correct answer (6 grams), then it was counted as having salt-reduction knowledge. With respect to salt-reduction practices, considering the main source of dietary sodium in China, which is added salt and soy sauce in home cooking, processed food and monosodium glutamate, eight specific questions were asked in the survey (mostly adopted from the WHO) [21]. These questions assess whether participants 1) limited their consumption of processed foods (yes or no); 2) paid attention to the salt (or sodium) content on food labels (yes or no); 3) reduced the frequency of eating out (yes or no); 4) put less salt in any food (yes or no); 5) ate less salt-rich foods, such as preserved foods, fermented bean curd, salted duck eggs, soybean paste, yellow sauce, etc. (yes or no); 6) did not add extra salt during eating (yes or no); 7) used a salt-control spoon (yes or no); and 8) used low-sodium salt (yes or no) [22]. If the respondent answered yes to any one of the eight specific questions, they were considered to engage in salt-reduction practice.

In China, salt-control spoons are a salt-reduction method designed to limit salt consumption during home cooking, and there are several sizes of salt-control spoons. There are usually 6-g size or 3-g size or 2-g size salt-control spoons selling in the market. The design of the salt-control spoons was to help the Chinese residents to be more realize how much salt they could intake for each day and help them to control their salt intake below the maximum of salt intake recommended from the Chinese Dietary Guidelines if they were using them in a correct way, not taking salt exceed the edge the spoons. The salt-control spoons are like the powder spoons, which do not have the scales. If the salt was not taken higher than the edge of the spoons, it means the salt was not taken than the maximum of size the salt-control spoons. For example, the 6-g size of salt-control spoon means it can hold out maximum of 6 grams salt if the salt is not higher than the edge of the spoon. Salt-control spoons were allocated to residents in several provinces of China as a part of salt-reduction campaigns or other salt-reduction related studies, and they can also be purchased from markets. Low-sodium salt was recommended by the WHO for consuming less sodium from salt and can be purchased from markets in China. The efficacy of the use of salt-control spoons and low-sodium salt has been studied and proven in China [23–25]. Furthermore, the main source of sodium consumption in China is home cooking [26]. Thus, a separate analysis was conducted by focusing on these two salt-reduction practices.

#### Anthropometry

Weight and height were measured in light clothing and without shoes using a unified electronic scale (G&G TC-150KA) and sitting height meter (TZG) on flat and firm land. Body Mass Index (BMI) was calculated by height (cm)/weight^2^ (kg). Obesity was defined as BMI ≥ 30 kg/m^2^; overweight was defined as 25 kg/m^2^ ≤ BMI <30 kg/m^2^ [13].

### Other medical conditions

Participants reported whether they had diagnosed diabetes, myocardial infarction, and stroke.

### Statistical analysis

After excluding missing data of related analyzed variables in this study, the final sample size was 179,834. Hypertension prevalence, percentages of salt-reduction knowledge and practice in each subgroup were calculated with stratification, clustering and sample weights [15]. The chi-square test was calculated with the mentioned prevalence or percentage of each subgroup to present the statistical significance level of the results. Multivariate hierarchical logistic regression was used to determine the relationship between hypertension and two specific salt-reduction practices (salt-control spoon and low-sodium salt) with ORs and 95% confidence intervals (CIs) by adjusting for related confounders. Variables that were significantly associated with salt-reduction practice at *P* < 0.05 in univariate analysis were then entered into multivariate modeling through hierarchical regression. Two multivariable logistic regression models were built. Model one was built with variables of basic demographic information. Model two was adjusted for basic demographic information, BMI, salt-reduction knowledge, and self-reported diabetes, myocardial infarction and stroke. All analyses in this study were conducted by SAS (version 9.4, SAS Institute Inc., Cary, NC, USA). The graphs were generated by R software (version 4.1.3).

## Results

The characteristics of the study participants are presented in Table 1. Out of 179,834 participants included in the analysis, 51.7% were female; the mean age was 43.9 (SD 14.4). A total of 8.1% reported a previous diagnosis of hypertension (established hypertension), 21.2% were newly diagnosed with hypertension, and 70.7% did not have hypertension (Table 1).

**Table 1:** Sociodemographic and anthropometry characteristics of the Chinese adults in 2015–16 (%).

		Hypertension in overall (*n* = 179,834)	Established hypertension (*n* = 23,061)	Newly diagnosed hypertension (*n* = 48,531)	No hypertension (*n* = 108,242)	*p* value
Total		29.3	8.1	21.2	70.7	**<0.0001**
*p* value			**<0.0001**			
Chi-square value			**559.3**			
Age group						
18–44 years		24.0	8.2	30.0	66.1	**<0.0001**
45–59 years		35.2	33.7	35.8	23.8	**<0.0001**
≥60 years		40.8	58.1	34.2	10.1	**<0.0001**
*p* value (trend test)		**<0.0001**	**<0.0001**	**<0.0001**	**<0.0001**	
t value (trend test)		**30.14**	**26.1**	**15.13**	**−30.14**	
Sex						
Male		54.6	46.6	57.7	48.4	**<0.0001**
Female		45.4	53.4	42.3	51.6	**<0.0001**
*p* value		**<0.0001**	**<0.0001**	**<0.0001**	**<0.0001**	
Education						
Primary or less		49.8	53.5	48.4	29.0	**<0.0001**
Secondary school		30.2	26.7	31.5	34.9	**<0.0001**
High school		13.3	13.7	13.1	18.3	**<0.0001**
College and above		6.8	6.1	7.0	17.8	**<0.0001**
*p* value (trend test)		**<0.0001**	**<0.0001**	**<0.0001**	**<0.0001**	
t value (trend test)		**−34.29**	**−40.43**	**−39.66**	**34.29**	
Living area						
Urban		47.3	54.2	44.6	53.1	**<0.0001**
Rural		52.7	45.8	55.4	46.9	**<0.0001**
*p* value		**<0.0001**	**0.0494**	**<0.0001**	**<0.0001**	
Living region						
North		52.5	50.4	53.3	44.9	**<0.0001**
South		47.5	49.6	46.7	55.1	**<0.0001**
*p* value		**<0.0001**	**0.0799**	**<0.0001**	**<0.0001**	
Occupation						
physical working population		61.9	50.5	66.3	61.1	**<0.0001**
nonphysical working population		8.8	7.7	9.2	16.3	**<0.0001**
students and sodier		0.4	0.3	0.5	2.4	**<0.0001**
others		28.9	41.6	24.0	20.2	**<0.0001**
*p* value		**<0.0001**	**<0.0001**	**<0.0001**	**<0.0001**	
Provinces (have salt reduction campaigns)						
no		49.4	51.4	48.6	43.9	**<0.0001**
yes		50.6	48.6	51.4	56.1	**<0.0001**
*p* value		**0.0003**	**0.0006**	**0.0148**	**0.0003**	
Body weight						
normal and low body weight		47.0	41.5	49.2	69.8	**<0.0001**
overweight		41.3	45.3	39.7	25.7	**<0.0001**
obesity		11.7	13.3	11.1	4.5	**<0.0001**
*p* value		**<0.0001**	**<0.0001**	**<0.0001**	**<0.0001**	

The physical working population included the production staff of agriculture, forestry, animal husbandry, fishery and water conservancy, production and transportation equipment operators and related personnel, and other physical workers.

The nonphysical working population included commercial and service workers, heads of state organs, party and mass organizations, enterprises and institutions, office staff and related personnel, and professional skilled workers.

Others were the home-cooking population.

*P* value and Chi-square values are shown in bold values, aiming to distinguish between the results and to highlight the results (results are statistical significant).

### Salt-reduction knowledge

**Fig. 1:**
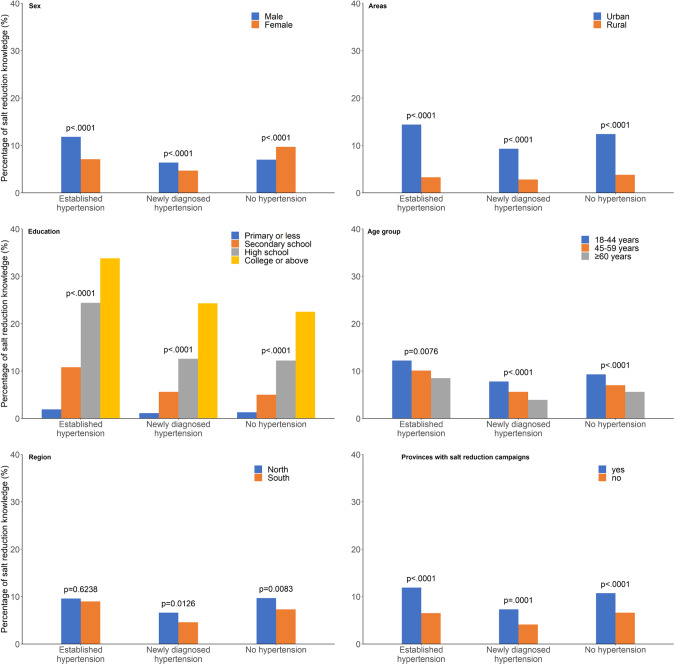
The percentage of salt-reduction knowledge. The weighted percentages of salt-reduction knowledge were distributed among three hypertension groups (established, newly diagnosed and no hypertension) in subgroups of sex, living areas (urban and rural areas), education level, age groups, regions (north and south) and provinces with salt-reduction campaigns.

Only 7.9% of participants had salt-reduction knowledge. The established hypertension group had the highest salt-reduction knowledge (9.2%), followed by the no hypertension group (8.2%) and the newly diagnosed hypertension group (5.4%). The salt-reduction knowledge in the established hypertension group was higher than that in the no hypertension group (*P* = 0.0963), but the percentage in the newly diagnosed hypertension group was significantly lower than that in the no hypertension group (*P* < 0.0001). This trend was also found in other subgroups, except for the education and occupation subgroups (Fig. 1).

Males had a lower level of salt-reduction knowledge than females (*P* < 0.0001). However, in the established hypertension and newly diagnosed hypertension groups, males had a significantly higher level of salt-reduction knowledge than females (P < 0.0001 in both groups). People living in urban areas had a significantly higher level of salt-reduction knowledge than rural areas in all hypertension groups (Fig. 1).

As the level of education increased, salt-reduction knowledge significantly increased in all hypertension groups (*P* < 0.0001). The level of salt-reduction knowledge in the age group ≥60 years was the lowest, and the level of knowledge was higher among people living in the north than among those living in the south in all groups. People living in provinces where salt-reduction campaigns had been implemented before 2015 had a higher level of salt-reduction knowledge than those living in provinces where campaigns were not implemented before 2015 (*P* < 0.0001) (Fig. 1).

### Salt-reduction practice

**Fig. 2:**
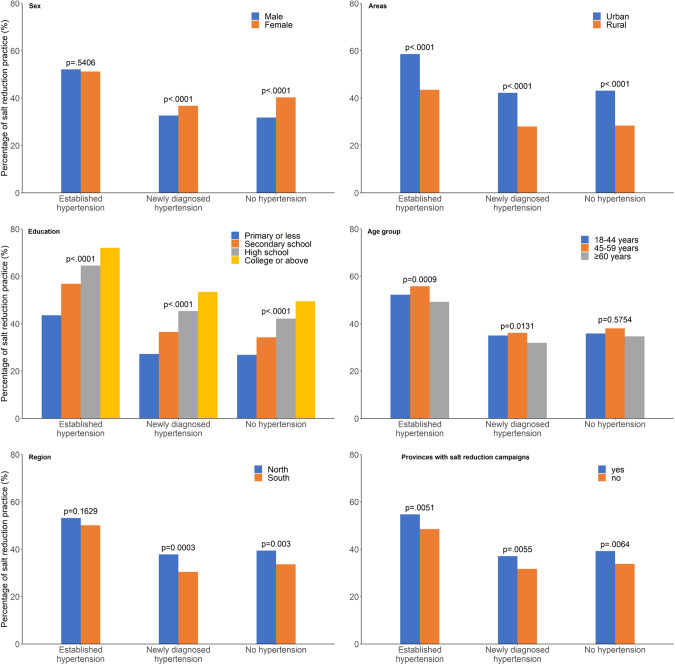
The percentage of salt-reduction practice. The weighted percentages of salt-reduction practice were distributed among three hypertension groups (established, newly diagnosed and no hypertension) in subgroups of sex, living areas (urban and rural areas), education level, age groups, regions (north and south) and provinces with salt-reduction campaigns.

Among all participants, the percentage of salt-reduction practice was 37.1%. The percentage was the highest in the established hypertension group (51.7%) and the lowest in the newly diagnosed hypertension group (36.2%) (Table 2). The percentage of salt-reduction practice in the established hypertension group was statistically significantly higher than that in the no hypertension group (*P* < 0.0001), but the salt-reduction practice in the newly diagnosed hypertension group was statistically significantly lower than that in the no hypertension group (*P* < 0.0001). The other subgroups showed similar trends (Fig. 2).

**Table 2:** percentage of eight salt-reduction practice (%).

	Total (*n* = 179,834)	Established hypertension (*n* = 23,061)	Newly diagnosed hypertension (*n* = 48,531)	No hypertension (*n* = 108,242)
Salt-reduction practice				
Yes	37.1	51.7	34.3	36.2
No	62.9	48.3	65.7	63.8
Limited consumption of processed foods				
Yes	22.0	31.3	18.9	21.8
No	78.0	68.7	81.1	78.2
Paid attention to the salt (or sodium) content on the food label				
Yes	13.3	18.5	11.5	13.2
No	86.7	81.5	88.5	86.8
Reduced eating out				
Yes	22.6	32.1	19.7	22.5
No	77.4	67.9	80.3	77.5
Put less salt in any food				
Yes	35.4	49.9	33.0	34.5
No	64.6	50.1	67.0	65.5
Ate less salt-rich foods, such as preserved foods, fermented bean curd, salted duck eggs, soybean paste, yellow sauce, etc.				
Yes	27.8	40.3	24.8	27.2
No	72.2	59.7	75.2	72.8
Did not add extra salt during eating				
Yes	25.3	35.7	22.2	25.0
No	74.7	64.3	77.8	75.0
Used a salt-control spoon				
Yes	10.7	16.1	9.6	10.4
No	89.3	83.9	90.4	89.6
Used low-sodium salt				
Yes	12.2	16.0	10.1	12.3
No	87.8	84.0	89.9	87.7

Males had a significantly lower percentage of salt-reduction practice than females (*P* < 0.0001). Males and females had similar levels of salt-reduction practice in the established hypertension groups (*P* = 0.5406), but the percentage among males was slightly lower than that of females in the newly diagnosed hypertension group (*P* < 0.0001) and the no hypertension group (*P* < 0.0001). Across all hypertension groups, people living in an urban area had a statistically significantly higher percentage of salt-reduction practice than those living in a rural area (*P* < 0.0001). As the education level increased, the percentage of salt-reduction practice also increased across all hypertension groups (*P* < 0.0001). The percentage of salt-reduction practice in the age group ≥60 years was the lowest across all hypertension groups. People living in the north had a higher percentage of salt-reduction practice than those living in the south across all hypertension groups (*P* = 0.0016). People living in provinces where salt-reduction campaigns had been implemented before 2015 had a significantly higher percentage of salt-reduction practice than provinces that had not implemented campaigns before 2015 (*P* = 0.0025) (Fig. 2).

### Use of salt-control spoons

The percentage of using salt-control spoons was 10.7%. The percentage was highest in the established hypertension group (16.1%), followed by the no hypertension group (10.4%) and newly diagnosed hypertension group (9.6%) (*P* < 0.0001) (Table 2).

**Fig. 3:**
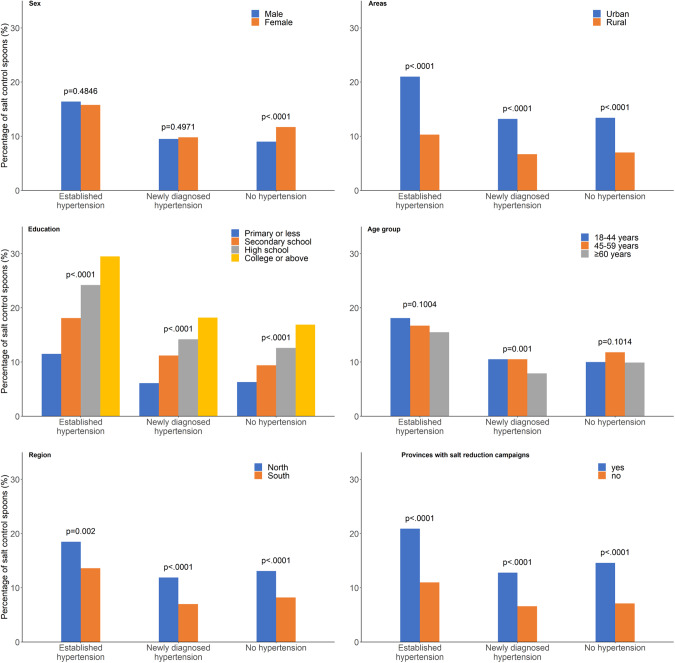
The percentage of using salt-control spoons. The weighted percentages of salt-control spoons were distributed among three hypertension groups (established, newly diagnosed and no hypertension) in subgroups of sex, living areas (urban and rural areas), education level, age groups, regions (north and south) and provinces with salt-reduction campaigns.

Males had a lower percentage of using salt-control spoons than females (*p* < 0.0001). The use of salt-control spoons in rural areas was statistically significantly lower than that in urban areas (*P* < 0.0001). In the education group, the use of salt-control spoons increased with increasing education level (*P* < 0.0001), and in the age group ≥60 years, it was slightly higher than in youngest age group (18–44 years), but slightly lower than the middle age group (45–49 years). People living in the north had a statistically significantly higher percentage of using salt-control spoons than those living in the south (*P* < 0.0001). People living in provinces where salt-reduction campaigns had been implemented before 2015 had a significantly higher percentage of using salt-control spoons than those living in provinces that did not implement campaigns before 2015 (*P* < 0.0001) (Fig. 3).

### Use low-sodium salt

**Fig. 4:**
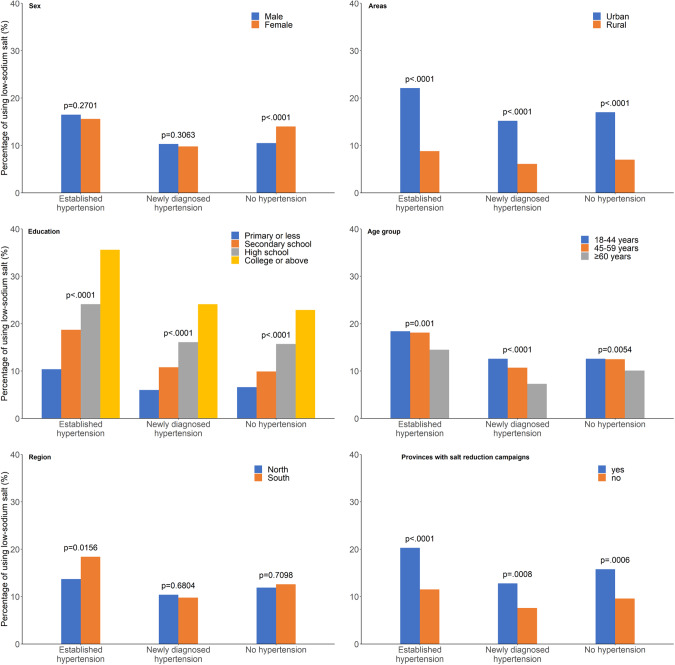
The percentage of using low-sodium salt. The weighted percentages of low-sodium salt were distributed among three hypertension groups (established, newly diagnosed and no hypertension) in subgroups of sex, living areas (urban and rural areas), education level, age groups, regions (north and south) and provinces with salt-reduction campaigns.

Overall, the percentage of using low-sodium salt was 12.2%. The highest percentage was in the established hypertension group (16%), followed by the no hypertension group (12.3%) and the newly diagnosed hypertension groups (10.1%) (Table 2). Males had a lower percentage of using low-sodium salt than females (*P* < 0.0001). The use of low-sodium salt in rural areas was low in all hypertension groups and statistically significantly lower than in urban areas (*P* < 0.0001). In the education group, the use of low-sodium salt increased with increasing education level (*P* < 0.0001), and in the age group ≥60 years, it was the lowest across all hypertension groups. People living in the south had a slightly higher percentage of using low-sodium salt than those living in the north (*P* = 0.6483). People living in provinces where salt-reduction campaigns had been implemented before 2015 had a significantly higher percentage of using low-sodium salt than those living in provinces that had not implemented campaigns before 2015 (*P* = 0.0002) (Fig. 4).

### Association between hypertension and the use of salt-control spoons

Females in the established hypertension groups (OR = 1.18; 95% CI: 1.06–1.32) and newly diagnosed hypertension group (OR = 1.23; 95% CI: 1.10–1.36) were more likely to use salt-control spoons than males, and people living in rural areas in the established hypertension group (OR 0.58; 95% CI: 0.44–0.77) and newly diagnosed hypertension group (OR 0.64; 95% CI: 0.51–0.81) were less likely to use salt-control spoons than people living in urban areas. People living in south in the established (OR 0.63; 95% CI: 0.51–0.78) and newly diagnosed (OR 0.60; 95% CI: 0.47–0.77) hypertension groups were less likely to use salt-control spoons than people living in the north, and people living in provinces having salt-reduction campaigns before 2015 were more likely to use salt-control spoons in the established (OR 1.98; 95% CI: 1.61–2.42) and newly diagnosed (OR 1.90; 95% CI: 1.49–2.42) hypertension groups. Having salt-reduction knowledge was significantly associated with a higher likelihood of using salt-control spoons in the established (OR 3.60; 95% CI: 3.00–4.32) and newly diagnosed (OR 3.91; 95% CI: 3.25–4.70) hypertension groups (Table 3).

**Table 3:** Odds ratio of using salt-control spoons among Chinese adults.

	Unadjusted (Univariate analysis)		Model 1		Model 2	
	Established hypertension	Newly diagnosed hypertension	Established hypertension	Newly diagnosed hypertension	Established hypertension	Newly diagnosed hypertension
	OR (95% CI)	OR (95% CI)	OR (95% CI)	OR (95% CI)	OR (95% CI)	OR (95% CI)
Sex						
Male	1.00	1.00	1.00	1.00	1.00	1.00
Female	0.96 (0.85–1.08)	1.03 (0.94–1.14)	1.19 (1.07–1.32)	1.26 (1.13–1.40)	1.18 (1.06–1.32)	1.23 (1.10–1.36)
Areas						
Urban	1.00	1.00	1.00	1.00	1.00	1.00
Rural	0.43 (0.34–0.55)	0.47 (0.37–0.59)	0.54 (0.41–0.73)	0.61 (0.48–0.77)	0.58 (0.44–0.77)	0.64 (0.51–0.81)
Region						
North	1.00	1.00	1.00	1.00	1.00	1.00
South	0.70 (0.56–0.87)	0.56 (0.43–0.72)	0.65 (0.53–0.80)	0.58 (0.45–0.74)	0.63 (0.51–0.78)	0.60 (0.47–0.77)
Age groups						
18–44 years	1.00	0.99	1.00	1.00	1.01	1.00
45–59 years	0.91 (0.67–1.23)	1.01 (0.87–1.17)	0.96 (0.69–1.33)	1.18 (1.01–1.37)	0.95 (0.69–1.29)	1.15 (0.99–1.34)
≥60 years	0.83 (0.62–1.10)	0.73 (0.60–0.88)	1.09 (0.79–1.51)	1.04 (0.84–1.28)	0.99 (0.72–1.36)	1.00 (0.80–1.24)
Education						
Primary or less	1.00	1.00	1.00	1.00	1.00	1.00
Secondary school	1.71 (1.49–1.95)	1.94 (1.69–2.23)	1.47 (1.28–1.68)	1.69 (1.44–1.98)	1.25 (1.09–1.44)	1.51 (1.29–1.76)
High school	2.46 (2.06–2.95)	2.55 (2.15–3.01)	1.88 (1.59–2.23)	1.88 (1.50–2.36)	1.34 (1.12–1.60)	1.52 (1.21–1.91)
College and above	3.22 (2.32–4.48)	3.44 (2.57–4.60)	1.94 (1.40–2.68)	2.22 (1.51–3.28)	1.28 (0.97–1.69)	1.54 (1.02–2.32)
Occupation						
Physical working population	1.00	1.00	1.00	1.00	1.00	1.00
Nonphysical working population	2.77 (2.10–3.65)	2.45 (1.98–3.03)	1.63 (1.27–2.10)	1.56 (1.23–1.98)	1.42 (1.13–1.78)	1.37 (1.05–1.78)
Students and soldiers	1.39 (0.77–2.51)	1.28 (0.60–1.71)	0.84 (0.44–1.62)	0.70 (0.34–1.43)	0.98 (0.51–1.89)	0.63 (0.24–1.67)
Others	1.52 (1.28–1.80)	1.47 (1.24–1.73)	1.11 (0.88–1.39)	1.23 (1.01–1.49)	1.06 (0.84–1.33)	1.18 (0.97–1.44)
Provinces (have salt-reduction campaigns)						
No	1.00	1.00	1.00	1.00	1.00	1.00
Yes	2.15 (1.75–2.65)	2.08 (1.62–2.68)	2.04 (1.67–2.50)	1.99 (1.56–2.54)	1.98 (1.61–2.42)	1.90 (1.49–2.42)
BMI			–	–		
	1.01 (0.99–1.03)	1.06 (1.04–1.07)	–	–	0.98 (0.96–1.01)	1.01 (1.00–1.03)
Salt-reduction knowledge			–	–		
No	1.00	1.00	–	–	1.00	1.00
Yes	5.25 (4.30–6.40)	5.97 (4.90–7.29)	–	–	3.60 (3.00–4.32)	3.91 (3.25–4.70)
Diagnosed diabetes			–	–		
No	1.00	1.00	–	–	1.00	1.00
Yes	1.36 (1.19–1.56)	1.67 (1.36–2.06)	–	–	1.18 (1.03–1.35)	1.41 (1.15–1.74)
Diagnosed myocardial infarction			–	–		
No	1.00	1.00	–	–	1.00	1.00
Yes	1.24 (0.97–1.58)	0.81 (0.48–1.39)	–	–	1.19 (0.90–1.56)	0.74 (0.41–1.32)
Diagnosed stroke			–	–		
No	1.00	1.00	–	–	1.00	1.00
Yes	0.91 (0.76–1.09)	1.06 (0.79–1.42)	–	–	0.91 (0.76–1.09)	1.05 (0.79–1.40)

Module 1 was adjusted with sociodemographic variables; module 2 was adjusted with sociodemographic variables, salt-reduction knowledge and biological factors

### Association between hypertension and the use of low-sodium salt

Females in the established hypertension groups (OR = 1.26; 95% CI: 1.13–1.40) and newly diagnosed hypertension group (OR = 1.20; 95% CI: 1.07–1.36) were more likely to use low-sodium salt than males, and people living in rural areas in the established hypertension group (OR 0.50; 95% CI: 0.38–0.66) and newly diagnosed hypertension group (OR 0.50; 95% CI: 0.39–0.64) were less likely to use low-sodium salt than people living in urban areas. People living in the south in the established (OR 1.42; 95% CI: 1.10–1.82) and newly diagnosed (OR 1.03; 95% CI: 0.75–1.43) hypertension groups were more likely to use low-sodium salt than people living in the north, and people living in provinces having salt-reduction campaigns before 2015 were more likely to use low-sodium salt in the established (OR 1.67; 95% CI: 1.33–2.10) and newly diagnosed (OR 1.58; 95% CI: 1.17–2.12) hypertension groups. Having salt-reduction knowledge was significantly associated with higher odds of using low-sodium salt in the established (OR 2.67; 95% CI: 2.20–3.24) and newly diagnosed (OR 3.36; 95% CI: 2.80–4.04) hypertension groups (Table 4).

**Table 4:** Odds ratio of using low-sodium salt among Chinese adults.

	Unadjusted (Univariate analysis)		Model 1		Model 2	
	Established hypertension	Newly diagnosed hypertension	Established hypertension	Newly diagnosed hypertension	Established hypertension	Newly diagnosed hypertension
	OR (95% CI)	OR (95% CI)	OR (95% CI)	OR (95% CI)	OR (95% CI)	OR (95% CI)
Sex						
Male	1.00	1.00	1.00	1.00	1.00	1.00
Female	0.94 (0.83–1.05)	0.95 (0.85–1.05)	1.26 (1.13–1.41)	1.24 (1.09–1.40)	1.26 (1.13–1.40)	1.20 (1.07–1.36)
Areas						
Urban	1.00	1.00	1.00	1.00	1.00	1.00
Rural	0.34 (0.26–0.44)	0.36 (0.29–0.45)	0.48 (0.36–0.63)	0.48 (0.37–0.61)	0.50 (0.38–0.66)	0.50 (0.39–0.64)
Region						
North	1.00	1.00	1.00	1.00	1.00	1.00
South	1.41 (1.07–1.87)	0.93 (0.66–1.30)	1.43 (1.11–1.83)	0.98 (0.71–1.35)	1.42 (1.10–1.82)	1.03 (0.75–1.43)
Age groups						
18–44 years	1.00	1.00	1.00	1.00	1.00	1.00
45–59 years	0.98 (0.71–1.34)	0.83 (0.72–0.95)	1.10 (0.80–1.50)	1.03 (0.90–1.17)	1.08 (0.80–1.47)	1.01 (0.89–1.16)
≥60 years	0.75 (0.56–1.01)	0.54 (0.47–0.63)	1.05 (0.74–1.47)	0.83 (0.71–0.96)	0.95 (0.67–1.35)	0.82 (0.70–0.95)
Education						
Primary or less	1.00	1.00	1.00	1.00	1.00	1.00
Secondary school	1.98 (1.71–2.29)	1.88 (1.59–2.23)	1.75 (1.47–2.09)	1.60 (1.29–1.97)	1.56 (1.30–1.86)	1.45 (1.18–1.78)
High school	2.73 (2.16–3.45)	2.99 (2.40–3.72)	2.08 (1.60–2.70)	2.18 (1.65–2.89)	1.61 (1.24–2.09)	1.82 (1.37–2.43)
College and above	4.76 (3.44–6.57)	4.93 (3.63–6.70)	3.04 (2.13–4.34)	2.95 (1.95–4.46)	2.27 (1.61–3.20)	2.19 (1.42–3.37)
Occupation						
Physical working population	1.00	1.00	1.00	1.00	1.00	1.00
Nonphysical working population	3.25 (2.52–4.20)	2.57 (2.05–3.23)	1.49 (1.19–1.85)	1.22 (0.97–1.53)	1.34 (1.08–1.66)	1.07 (0.84–1.36)
Students and soldiers	1.60 (0.54–4.81)	1.33 (0.58–3.06)	0.78 (0.27–2.21)	0.58 (0.26–1.34)	0.87 (0.30–2.50)	0.51 (0.17–1.54)
Others	1.61 (1.32–1.97)	1.26 (1.02–1.54)	1.12 (0.89–1.42)	1.05 (0.84–1.31)	1.08 (0.85–1.37)	1.01 (0.81–1.27)
Provinces (have salt-reduction campaigns)						
No	1.00	1.00	1.00	1.00	1.00	1.00
Yes	1.95 (1.50–2.53)	1.77 (1.29–2.42)	1.72 (1.37–2.17)	1.65 (1.23–2.23)	1.67 (1.33–2.10)	1.58 (1.17–2.12)
BMI			–	–		
1.00(0.99–1.02)		1.06 (1.04–1.07)	–	–	0.99 (0.97–1.00)	1.02 (1.00–1.04)
Salt-reduction knowledge						
No	1.00	1.00			1.00	1.00
Yes	4.46 (3.59–5.54)	5.58 (4.54–6.87)			2.67 (2.20–3.24)	3.36 (2.80–4.04)
Diagnosed diabetes						
No	1.00	1.00			1.00	1.00
Yes	1.31 (1.11–1.55)	1.28 (0.99–1.65)			1.13 (0.97–1.31)	1.18 (0.89–1.56)
Diagnosed myocardial infarction			–	–		
No	1.00	1.00	–	–	1.00	1.00
Yes	1.32 (1.06–1.66)	0.58 (0.31–1.07)	–	–	1.43 (1.12–1.82)	0.67 (0.36–1.24)
Diagnosed stroke			–	–		
No	1.00	1.00	–	–	1.00	1.00
Yes	0.80 (0.66–0.96)	0.77 (0.60–0.97)	–	–	0.91 (0.76–1.08)	0.92 (0.72–1.18)

Module 1 was adjusted with sociodemographic variables; module 2 was adjusted with sociodemographic variables, salt-reduction knowledge and biological factors

## Discussion

This is the first nationally representative study evaluating salt-reduction knowledge and practice in China. We sought to determine the percentage of salt-reduction knowledge and practice in established hypertensive, newly diagnosed hypertensive and no hypertensive population at the national scale and how regions (north and south), areas (urban and rural), provinces (having salt-reduction campaigns before and not), sex, and age were associated with salt-reduction knowledge and practice in hypertension groups. Our work highlights the need to promote education related to hypertension, salt-reduction knowledge and practice among the public as well as the need to strengthen strategies of popularization of salt-reduction knowledge and practice among males, rural areas, northern regions and aging populations in China. At the same time, it is worth noting that more than 2 in 3 people with hypertension were not diagnosed. This implies that synergistically screening for hypertension and promoting salt-reduction practices could be a way forward.

The results from this study confirmed that conducting salt-reduction campaigns in provinces of China is related to more salt-reduction knowledge and practice in the provinces, which is consistent with other studies [27, 28]. Conducting salt-reduction campaigns promotes the generalization of salt-reduction knowledge and behaviors. This study found that populations with salt-reduction knowledge were more than two or even three times more likely to use salt-control spoons and low-sodium salt, thus emphasizing the importance of salt-reduction knowledge to salt-reduction behaviors, which is consistent with the results from other studies [29–31].

Our study found that the percentages of having salt-reduction knowledge and practice were low in the population, especially in the newly diagnosed hypertension population, which accounted for a significantly larger proportion in the hypertension population, suggesting that it is much necessary to strengthen the popularization and application of salt-reduction knowledge and practices to the public. The higher knowledge among the established hypertensive population might be due to the opportunity to interact with health care providers. The second reason might be that the hypertensive group could have more willingness than no hypertension group to increase their awareness and change their lifestyle practices [32]. Regarding the newly diagnosed hypertension population, after the diagnosis, they had the recommendations for receiving medical services and treatment from the primary health care institutions in local community or nearest hospitals.

Interventions for salt-reduction need to be strengthened for the public, and strategies should focus more on tailored interventions in rural areas, males and older individuals. Despite the higher burden of hypertension (especially the newly diagnosed hypertension), the percentage of salt-reduction knowledge and practice was lower among these groups [33]. This could be due to the inequality of health resources, the demographic or the socioeconomic differences of the residents between rural and urban areas, especially the northern rural areas where residents tend to consume pickles in winter because of the poor availability of vegetables [34–39]. Meanwhile, this could also be due to the particular Chinese social and cultural context where women are mostly involved on food preparation and decision-making, including shopping food, leading males with lower salt-reduction knowledge and practice [40]. It needs be pointed out that older individuals prefer to take responsibilities in preparing meals at home, especially the older women, but older individuals in China have the lowest salt-reduction knowledge and practices among the age groups [40]. This could be because of the decline of cognition along with the age [41]. Promotion of salt-reduction knowledge and practices for older adults needs to take this into account. In any case, strengthening the promotion of salt-reduction knowledge and behaviors may lead to improved prevention and detection of hypertension [42].

In this study, we observed that people living in the north had more salt-reduction knowledge (9% in the north versus 6.9% in the south) and salt-reduction practice (40.2% in the north versus 34.3% in the south) than people living in the south, but the difference was not high, especially the salt-reduction knowledge. There are differences in food culture between the north and south in China, as the flavor of food in the north is saltier than that in the south [10]. In addition, northern China has a higher prevalence of hypertension [43]. To control hypertension prevalence in a targeted way, it is essential to emphasize limiting salt intake in northern China.

For the specific salt-reduction practice, we also observed that the use of low-sodium salt was low in both the north and south. Among the hypertensive group, the south had more possibilities for using low-sodium salt, but the north had more possibilities for using salt-control spoons, which may be due to the differences in publicity, acceptance and the supply size of low-sodium salt and salt-control spoons between the north and south. Using low-sodium salt is recommended by the WHO and has been proven to significantly reduce blood pressure [22–24], and using low-sodium salt can maintain a similar saline taste as food with less salt intake. However, as the region is favored with more salty food, using low-sodium salt to reduce blood pressure was not popularized in the northern region. This result indicates that the promotion of low-sodium salt needs to be strengthened, especially in northern China. The reason that the south of China can better accept low-sodium salt than the north needs further examined in the future. The use of salt-control spoons in this study was low, approximately 10.7%, which is slightly higher than the percentage (10.21%) in 2010 [44]. The reason behind this might be the inconvenience of using salt-control spoons due to the design shortage of salt-control spoons and the low distribution rate of salt-control spoons to residents [45]. Improving the design of the salt-control spoons and increasing promotion and education about the salt-control spoons and how to use it correctly to the public would promote the use the salt-control spoons and efficiently control the salt intake.

For the promotion of salt-reduction knowledge and practices, along with strengthening the current salt-reduction campaigns in China, there are some other national salt-reduction strategies that could be considered and combined in Chinese culture for adding more effectiveness, including food reformulation (under the salt target) and front of pack labeling with more nutritional information [46]. Besides, it needs to be strengthened to promote low-sodium salt in the market and salt-related education to the whole population. With a more complex environment in China than developed countries, multicomponent strategies for salt-reduction would be more effective.

This study has many strengths. First, the sample used in this study is large and representative at the national and provincial levels. Second, the response rate is high for all of the related variables, since the proportion of missing variables overall is 5.2%. Third, this survey used home-to-home visits for data collection to ensure that data were well collected. Furthermore, all the data collection was conducted by the interviewers with unified training and standard measuring tools. Nevertheless, this study has some limitations. Although due to the large sample size, the effect of the recall bias might not be significant, it cannot be ruled out for variables related to risk factors, salt-reduction knowledge and practices. Residual confounding was not completely eliminated because not all confounders were adjusted in the logistic regression model. There might be some hybrid factors in the region, and an ecological fallacy might exist in the outcomes of this study. The outcome of this study cannot be explained at the individual level. As we used the third version of Chinese Dietary Guidelines (2007) (salt target was 6 grams) as the standard for categorizing having salt-reduction knowledge, which standard was continuous using during the survey. If participants answered 5 grams recommended by WHO guideline, they were not categorized as having salt-reduction knowledge.

In conclusion, our research results show that there is a need to increase salt-reduction knowledge and practice across China, with higher emphasis among the northern region, rural areas, males, and the aging population. Increasing the promotion of the low-sodium salt and salt-control spoons is also needed. Considering that more than 70% of people with hypertension were undiagnosed in 2015, synergistically screening for hypertension in promoting salt-reduction practices may be a way forward. Under the complex environment in China, multicomponent strategies for salt-reduction knowledge and practices could be more effective. Although the results were from China, salt-reduction knowledge and practice may be similar in other low- and middle-income countries with similar sociodemographic transitions.

## Summary

### What is known about this topic

- Reducing salt intake can lower blood pressure and the amount of salt consumed by the Chinese in 2020 (9.3 grams) was still much higher than that recommended by the Chinese government in 2015(6 grams). Therefore, it is necessary to let the public, especially hypertensive patients, understand the importance of reducing salt intake.
- China’s “Action on Salt China” conducted from 2017 to 2021 was held in six provinces in China, aiming to increase salt-reduction knowledge and practices.
- It is necessary to study the national salt-reduction knowledge and behavior in China after the campaign, because it can help us understand the current national rate of salt-reduction knowledge and behavior, and provide an effective premise for the government’s further salt-reduction intervention measures.
- There are few national studies on salt-reduction knowledge and behavior.

### What this study adds

- This study can reflect the popularity of salt-reduction knowledge and behavior nationwide in China.
- This paper also studied salt-reduction knowledge and behaviors in subpopulations and hypertension populations, to provide the rate of salt-reduction knowledge and behaviors in different populations. Therefore, this study can provide a research basis for the China government to customize salt-reduction knowledge and behavioral intervention policies for different population groups in China.

## Data Availability

The data used in this study is available from the National Center for Chronic and Non-Communicable Disease Control and Prevention, Chinese Center for Disease Control and Prevention.
